# Prognostic significance of co-expression of c-MET and EGFR in locally advanced and metastatic gastric cancer: a retrospective cohort study

**DOI:** 10.3389/fonc.2026.1936498

**Published:** 2026-09-24

**Authors:** Zhang Ying, Wang Xiaoli, Liu Xiaoyuan, Shen Cailu, Shen Di, Ge Xiaosong

**Affiliations:** 1 Department of Medical Oncology, Affiliated Hospital of Jiangnan University, Wuxi, Jiangsu, China; 2 Pathology Department, Affiliated Hospital of Jiangnan University, Wuxi, Jiangsu, China; 3 Pharmacy Department, Affiliated Hospital of Jiangnan University, Wuxi, Jiangsu, China

**Keywords:** c-MET, co-expression, EGFR, gastric cancer, prognosis

## Abstract

**Objective:**

Gastric cancer (GC) remains a leading cause of cancer-related mortality globally. While molecular targeted therapies against HER2 and PD-L1 have advanced, the role of receptor tyrosine kinase (RTK) co-activation, specifically between c-MET and EGFR, remains elusive in GC. This study aimed to investigate the correlation between c-MET and EGFR overexpression and clinicopathological features and prognosis in patients with gastric cancer, and to reveal prognostic significance of co-expression of c-MET and EGFR in gastric adenocarcinoma.

**Methods:**

The clinicopathological data of 214 Chinese patients with GC were retrospectively analyzed, and the expression level of c-MET and EGFR were detected by immunohistochemistry. χ^2^ test was used to investigate associations with clinicopathological features. Kaplan-Meier survival curve and Cox proportional hazards models were employed to analyze the relationship between c-MET, EGFR and co-expression and patient prognosis.

**Results:**

The positive expression rates for c-MET and EGFR were 21.96% (47/214) and 68.22% (146/214), respectively. c-MET overexpression was significantly served as an independent predictor of poor OS (*P=0.023*). Conversely, EGFR expression alone showed no significant prognostic value. Notably, 18.22% (39/214) of cases exhibited co-expression of c-MET and EGFR. Patients with isolated EGFR expression exhibited the most favorable prognosis, while the isolated c-MET expression group had the worst clinical survival. Moreover, patients with c-MET and EGFR co-expression achieved better survival than those with isolated c-MET expression (*P = 0.007*).

**Conclusion:**

c-MET overexpression is a robust prognostic indicator of adverse outcomes in GC. The frequent co-expression of c-MET and EGFR suggests a potential mechanism of RTK cross-talk, warranting further investigation into dual-targeted therapeutic strategies.

## Introduction

Gastric cancer (GC) is one of the common malignant digestive tumors and a leading cause of cancer-related mortality worldwide, with a particularly high burden in China (1). As emerging molecular novel targets been found in cancer, molecular targeted therapy have been evaluated in international randomized clinical trials for GC management (2). Prominent examples include human epidermal growth factor receptor type 2 (HER2) (3), fibroblast growth factor receptor (FGFR) (4), programmed death-ligand 1(PD-L1) (5), claudin18.2 (CLDN18.2) (6). Despite these advancements, there remains an unmet clinical need for additional targeted agents to improve patient outcomes.

c-Mesenchymal-epithelial transition (c-MET) is a member of the receptor tyrosine kinase (RTK) family, abnormal activation of which drives malignant tumor cells division, proliferation and invasion (7). Recent studies found that MET signaling pathway has been associated with poor clinical outcomes in GC, highlighting the therapeutic potential of c-MET (8). Although numerous anti-tumor agents targeting c-MET have been developed, their clinical efficacy has been limited by intrinsic resistance and adverse events (9–11).

“RTK coactivation” constitutes a critical mechanism by which cancer cells utilize to evade normal cellular processing and clearance, fostering chemo-resistance and tumor progression (12). Functional cross talk between signaling pathways, particularly between epidermal growth factor receptor (EGFR), c-MET, exemplifies this interaction (13). Recent studies have identified concurrent mutations and protein co-expression of c-MET and EGFR across various malignancies, including breast (14), lung (15) and gastric (16, 17) cancer.

Consequently, dual blockade of EGFR and c-MET has emerged as a promising therapeutic strategy for GC. This approach posits that inhibiting co-activated network kinases may enhance efficacy and overcome resistance mechanisms. However, little is known about the co-expression patterns of c-MET and EGFR in Chinese human GC and its prognostic significance. In this study, we investigated the expression of c-MET and EGFR to determine whether they are useful as prognostic factors and therapeutic targets of gastric cancers, with a specific focus on the clinical implications of multiple RTK co-expression.

## Materials and methods

### Patients

A total of 214 locally advanced and metastatic gastric cancer patients with definite pathological diagnosis in the Affiliated Hospital of Jiangnan University were collected in this study from August 2022 to August 2023. Clinical data collected, include sex, age, tumor location, metastasis status, clinical stage, differentiated degree, vascular tumor thrombus presence, nerve infiltration, mismatch repair status (MMR), and the expression levels of PD-L1, HER2 and Ki-67. Survival follow-up was conducted until July 2024. TNM staging was published by the American Joint Committee on Cancer (AJCC) in 2019. This study was protocol approved by the Institutional Review Board of the Affiliated Hospital of Jiangnan University(LS2024311) and individual consent for this retrospective analysis was waived. The study inclusion criteria were as follows: (1) patients with gastric or gastroesophageal junction adenocarcinoma; (2) locally advanced and metastatic patients of III-IV TNM stage, excluding stage I-II; (3) patients who did or did not undergo systemic treatment. The exclusion criteria were as follows: (1) patients with other forms of gastric or gastroesophageal junction cancer except adenocarcinoma; (2) patients with other active malignancy; (3) patients with incomplete pathological information. Clinical trial number: not applicable.

### c-MET IHC and interpretation standards

Surgical resection or biopsy samples from eligible patients were cut into 4μm thick continuous sections and examined for c-MET protein. IHC analysis of c-MET was performed using the Ventana Benchmark ULTRA system (Roche Diagnostics) with c-MET primary rabbit antibody (clone GR524, Gene Tech, China, catalogue GT2374). Sections, dewaxing, repair, sealing, incubation of primary and secondary antibodies, horseradish peroxidase labeling, color rendering (DAB), counterstaining, dehydration, and sealing were performed successively. All slides were independently evaluated by two experienced pathologists who were blinded to the clinical data. In cases where the initial scores differed by more than one grade, the slides were re-examined simultaneously on a double-headed microscope until a consensus agreement was reached. The intensity of the c-MET was scored based on the four step scales and previously published criteria (16): score 0 was no reactivity or < 50% tumor cells with any membranous reactivity; score 1 was ≥50% with weak staining and <50% with moderate/strong staining; score 2 was ≥50% with moderate staining and <50% with strong staining; score 3 was ≥50% with strong staining. Among them, 0 to 1 is negative and 2 to 3 is positive.

### EGFR IHC and interpretation standards

EGFR IHC staining was also performed with Ventana kit instructions, using EGFR primary rabbit antibody (clone EP22, Gene Tech, China, catalogue GT2093). All slides were independently evaluated by two experienced pathologists who were blinded to clinical data. The consensus agreement consistent with that previously described. Lacking standard clinical criterion for EGFR determination, the intensity of the EGFR was also scored based on previously reported (16) as followed: score 0, no reactivity or membranous reactivity in less than 10% of tumor cells; score 1, faint/barely perceptible membranous reactivity in 10% or more of tumor cells or cells reactive only in part of their membrane; score 2, weak to moderate complete or basolateral membranous reactivity in 10% or more of tumor cells; and score 3, moderate to strong complete or basolateral membranous reactivity in 10% or more of tumor cells. EGFR positivity was defined as an IHC score of 2 or 3, while EGFR negativity was defined as an IHC score of 0 or 1.

### Statistical analysis

Statistical software SPSS 27.0 was used for data analysis. Associations between protein expression and clinicopathological variables were evaluated using theχ^2^ test. For biomarker analyses, no adjustment was made given their exploratory nature accordingly. Correlations between c-MET and EGFR expression were assessed via Spearman’s rank correlation coefficient. Overall survival (OS) was estimated using the Kaplan-Meier method and compared with the log-rank test. Multivariate survival analysis was conducted using the Cox proportional hazards model. A two-sided P<0.05 was considered statistically significant.

## Results

### c-MET immunohistochemical expression and clinicopathological features

Among the 214 cases, c-MET IHC scores were distributed as follows: 0 (n=80), 1+ (n=87), 2+ (n=42), and 3+ (n=5). The overall positive rate for c-MET was 20.41% (**Figures 1A–D**). As the patient distribution patterns for distant metastasis and TNM stage were comparable, these groups were merged into a single cohort for analysis. There was no association observed between c-MET expression and age, gender, tumor location, nerve invasion, differentiated degree, vascular tumor thrombus presence, metastasis status, lymph node metastasis status, DNA mismatch repair status (MMR), and the expression of PD-L1, HER2 and Ki-67(P > 0.05, **Table 1**).

**Figure 1:**
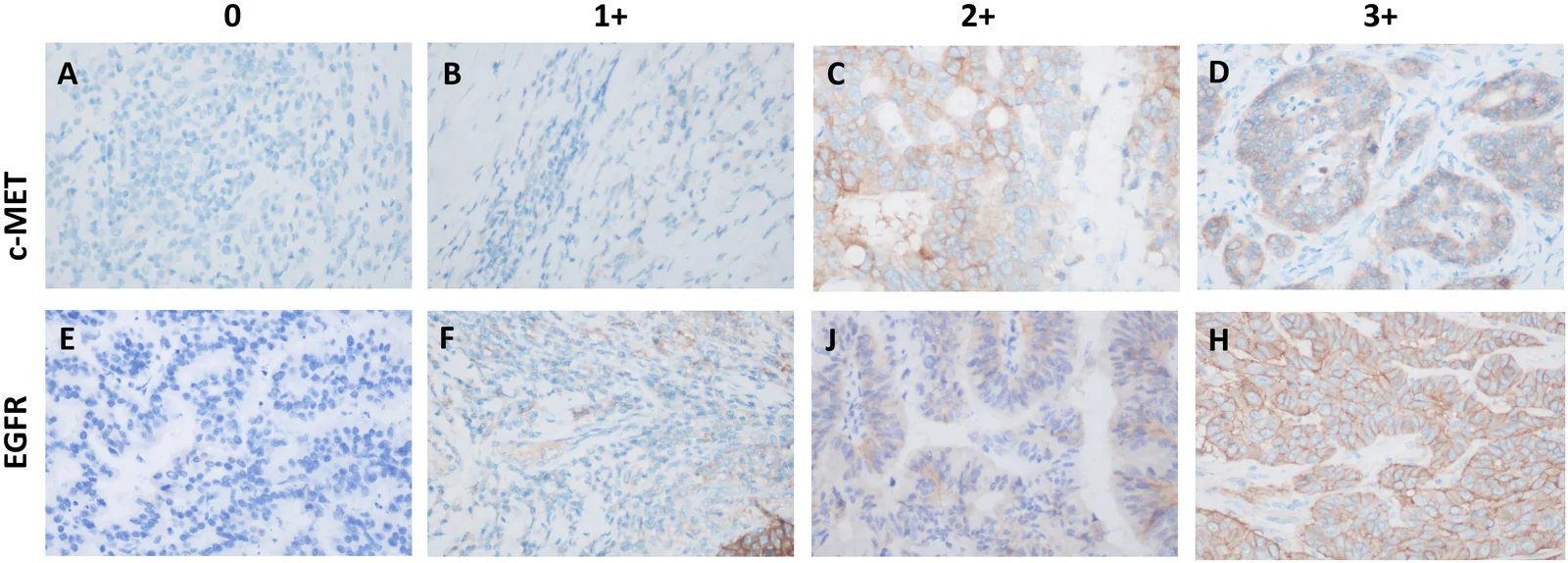
Representative figures of immunohistochemistry (IHC) scores for c-MET **(A–D)** and EGFR **(E–H)** expression in gastric cancers (original magnification 400×).

**Table 1:** c-MET expression and clinicopathological characteristics in patients with gastric cancer.

Clinicopathological characteristics	c-MET	c-MET	χ2	P	EGFR	EGFR	χ2	P
	Negative(n=167)	Positive (n=47)			Negative (n=68)	Positive (n=146)		
Age (years)			0.571	0.501			2.874	0.099
≤70	67	16			32	51		
>70	100	31			36	95		
Gender			0.059	0.808			2.315	0.147
Male	122	30			53	99		
Female	45	17			15	47		
Tumor location			0.170	0.739			0.597	0.461
Non antrum	98	26			42	82		
Antrum	69	21			26	64		
Nerve invasion			0.006	1.000			0.578	0.494
No	40	11			14	37		
Yes	127	36			54	109		
Tumor thrombus			1.275	0.300			0.025	1.000
No	61	13			23	51		
Yes	106	34			45	95		
Differentiation degree			0.338	0.669			0.126	0.705
Medium/high differentiation	40	7			13	25		
Low differentiation	136	40			55	121		
Lymph node metastasis			0.903	0.532			0.261	0.588
No	14	2			6	10		
Yes	153	45			62	136		
Distant metastasis/TNM stage			3.187	0.090			0.744	0.452
No/III	140	34			53	121		
Yes/IV	27	13			15	25		
PD-L1			0.019	1.000			2.707	0.235
Negative	126	35			55	106		
Positive	41	12			13	40		
Her-2			0.208	1.000			5.921	0.011*
0-1+	157	45			68	134		
2-3+	10	2			0	12		
dMMR			3.734	0.067			0.108	1.000
No	161	42			65	138		
Yes	6	5			3	8		
Ki67			0.107	0.850			1.686	0.237
≤60%	43	11			21	33		
>60%	124	36			47	113		

### EGFR immunohistochemical expression and clinicopathological features

Expression of EGFR was diffused in specimens score 2 and 3. Of the 214 specimens of gastric cancer, 146 (70.92%) were EGFR-positive (Score 2: n=116; Score 3: n=30, **Figures 1E, F**), while 114 (29.08%) were negative (Score 0: n=12; Score 1: n=56, **Figures 1J, H**). As detailed in **Table 1**, EGFR over-expression was significantly associated with low Her-2 protein expression (*P = 0.011*), whereas its correlation with other conventional clinicopathological features was not statistically significant (all *P > 0.05*, **Table 1**). Nevertheless, these findings warrant further validation in independent, larger patient cohorts.

### Correlation between c-MET and EGFR Expression

A significant positive correlation was observed between c-MET and EGFR protein expression (*P<0.05*, **Table 2**). No significant correlations were found between c-MET and other proteins. Combined analysis revealed that 39 patients (18.22%) exhibited concurrent overexpression of both c-MET and EGFR, 8 patients (3.73%) with c-MET overexpression alone, 107 patients (50.00%) with EGFR overexpression alone, and 60 patients (20.04%) with neither marker overexpressed.

**Table 2:** Correlation with c-MET.

Spearman correlation	Rs	P
EGFR	0.168	0.014*
PDL1	-0.027	0.690
HER-2	-0.031	0.650
dMMR	0.132	0.054
Ki-67	0.022	0.745

*P < 0.05 indicates a statistically significant difference.

### Impact of c-MET and EGFR expression on survival

OS was calculated from the date of initial diagnosis until death or the last follow-up (July 12, 2024). During these 2 years, 59 of the 214 enrolled patients (27.57%) succumbed to the disease, whereas 155 patients (72.43%) were alive and continued to be followed at the censor date, and 0 patient was lost to follow-up. Kaplan-Meier analysis demonstrated a significant difference in OS between c-MET-negative and c-MET-positive groups with c-MET positivity indicating poorer prognosis (**Figure 2A**, *P* = 0.023). In contrast, no correlation was observed between OS rate and EGFR positivity (**Figure 2B**, *P* = 0.227). As shown in **Table 3**, univariate Cox regression analysis demonstrated that c-MET overexpression, nerve invasion, tumor thrombus, and distant metastasis were each significantly correlated with worse OS (all *P <* 0.05). Variables with *P < 0.1* in univariate analysis were entered into the multivariate model. Multivariate Cox regression analysis confirmed nerve invasion and distant metastasis//TNM stage as an independent prognostic factor for OS. Meanwhile, c-MET status and tumor thrombus were not independently associated with poor OS in this specific multivariate model (**Table 3**, *P=0.099*). Regarding co-expression, Kaplan–Meier survival analysis revealed a statistically significant overall difference in cumulative survival among the four groups (c-MET-EGFR-, isolated c-MET+, isolated EGFR+, and c-MET+EGFR+, **Figure 2**, *P = 0.007*). Patients with isolated EGFR expression exhibited the most favorable prognosis, while the isolated c-MET expression group had the worst clinical survival. Moreover, patients with c-MET and EGFR co-expression achieved better survival than those with isolated c-MET expression, coexisting EGFR expression could partially offset the survival disadvantage conferred by c-MET overexpression.

**Figure 2:**
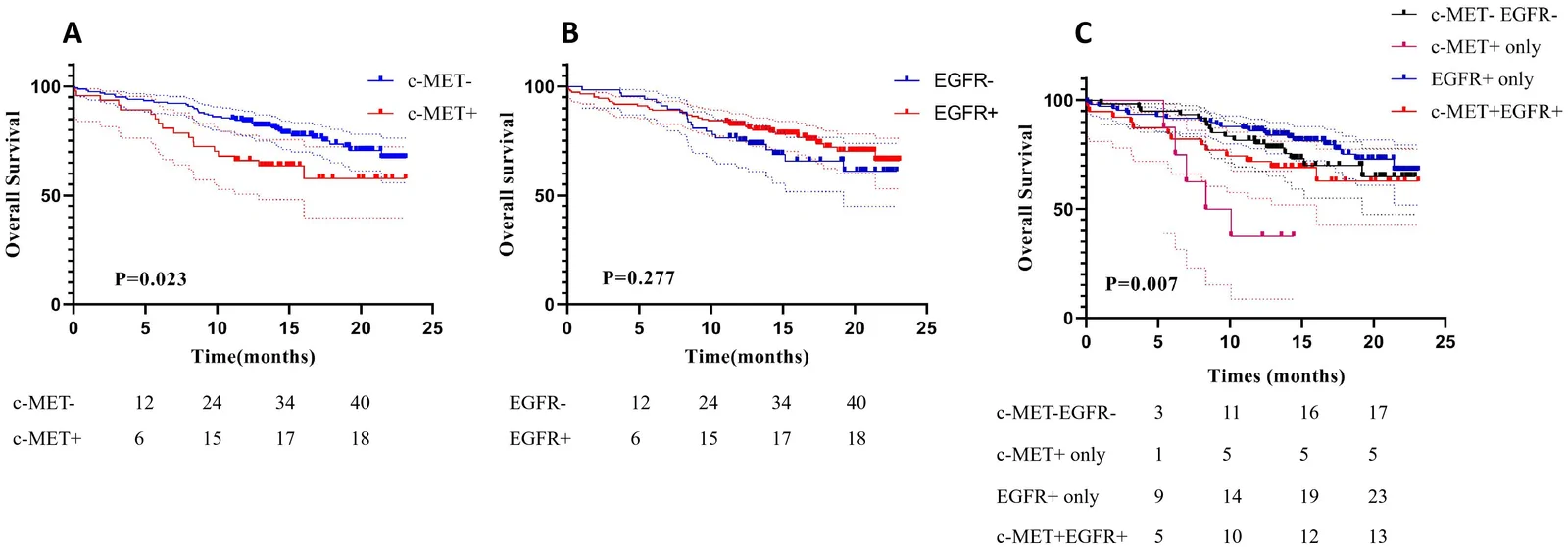
Kaplan-Meier survival curves of gastric cancer patients’ OS according to the c-MET **(A)**, EGFR **(B)** and co-expression of both **(C)**. The cumulative event counts at the respective time points are shown in the table beneath the survival plot.

**Table 3:** Univariate analysis and multivariate regression analysis of survival rate.

Variables	Single factor analysis		Multiple-factor analysis	
	P	HR (95% CI)	P	HR (95% CI)
c-MET	0.025*	0.531 (0.304-0.925)	0.099	1.606 (0.915-2.818)
EGFR	0.279	0.747 (0.440-1.267)	–	–
Co-expression of c-MET and EGFR	0.819	1.023 (0.844-1.239)		
Tumor location	0.604	1.146 (0.685-1.916)	–	–
Differentiation degree	0.173	0.578 (0.262-1.273)	–	–
Nerve invasion	0.013*	2.710 (1.229-5.974)	0.023*	2.507 (1.134-5.541)
Tumor thrombus	0.037*	1.895 (1.040-3.454)	0.078	1.719 (0.940-3.142)
Lymph node metastasis	0.131	4.585 (0.635-33.118)	–	–
Distant metastasis//TNM stage	<0.001*	3.626 (2.133-6.163)	<0.001*	3.432 (2.012-5.853)
MMR status	0.683	0.745 (0.182-3.056)	–	–
PD-L1	0.157	0.623 (0.323-1.200)	–	–
Her-2	0.149	0.233 (0.032-1.688)	–	–
Ki-67	0.454	0.806 (0.459-1.417)	–	–

*P < 0.05 indicates a statistically significant difference.

## Discussion

Cross-talk among RTKs facilitates tumor growth and metastasis, rendering them attractive candidates for combinatorial targeted therapy. As a key RTKs, c-MET has been implicated in accelerated tumor progression and therapeutic resistance across multiple cancer types, including lung, breast and gastric cancer (18). Emerging evidence highlights additional roles of c-MET in cancer through crosstalk with other receptors and cell surface proteins, especially with EGFRAs reported, the combined expression rate of EGFR and c-MET in colorectal cancer was significantly higher than the single-target expression rate, which also could identify metastatic lymph node tissue (19). In gastric adenocarcinoma, one study evaluated the over-expression of HER2, EGFR, MET and FGFR2, and found nearly 23% samples were positive for 2 or more RTKs (16). However, the study (16) did not specifically report the positive rate of both EGFR and c-MET expression, nor did it make any associations for the combination. In another small cohort study, both c-MET and c-EGFR overexpression were founded in 12(10%) Canadian GC patients, and associated with a trend toward poor OS (17). In the present study, we expanded the research scope and analyzed the level of c-MET and EGFR expression in Chinese GC patients to determine whether simultaneous changes can be used as therapeutic targets for better stratification and co-targeting therapy.

Of the 214 GC patients enrolled in this study, 21.96% (47/214) were identified as c-MET positive by immunohistochemistry, which is consistent with the positive rate reported in previous Chinese GC cohorts. Our results confirmed that c-MET overexpression was served as an independent predictor of poor OS in GC patients. Mechanistically, aberrant activation of c-MET can trigger multiple downstream oncogenic signaling pathways, including RAS/MAPK, PI3K/Akt and Wnt/β-catenin, which in turn promote GC cell invasion, metastasis and tumor stem cell maintenance (20). In recent years, several small-molecule TKIs targeting the HGF/c-MET pathway, such as sevotinib (21), crizotinib (22) and cabozantinib (23), have shown promising anti-tumor activity in preclinical and early clinical studies of c-MET-amplified GC. However, none of these drugs have been approved for GC treatment, mainly due to the lack of effective predictive biomarkers for patient stratification. Our study confirmed that c-MET protein overexpression is an independent poor prognostic marker in GC, which provides a potential biomarker for screening candidate patients who may benefit from c-MET-targeted therapy and lays a foundation for subsequent clinical trials of c-MET TKIs in GC.

EGFR normally participates in regulating cell proliferation, migration, and adhesion, which has become a research hotspot. Many studies have confirmed its positive expression in gastric cancer tissues. The positive expression rate of EGFR protein in this study was 68.22%(146/214), aligning with the broad range (8%-85%) documented in previous studies (24). EGFR positive expression is accompanied by HER2 expression in GC, which is consistent with previous studies (25). However, our data did not reveal a significant correlation between EGFR expression and other clinicopathological features. The possible reasons for this discrepancy are as follows: First, the inconsistency between EGFR protein expression and gene mutation/amplification status—EGFR prognostic value may depend on its genetic alteration rather than protein expression, and most GC patients with high EGFR protein expression lack EGFR gene mutation or amplification; Second, the heterogeneity of GC—our study included both intestinal and diffuse type GC, and the prognostic value of EGFR may vary with Lauren classification; Third, the lack of standardized IHC scoring criteria for EGFR in GC—at present, there is no unified IHC scoring standard for EGFR in GC, and different scoring methods may lead to different conclusions on its clinical significance. Further studies combining EGFR gene detection and standardized IHC scoring are needed to clarify the prognostic value of EGFR in GC.

Furthermore, we found that 39 patients (18.22%) showed positive co-expression of c-MET and EGFR, and there was a significant correlation between the expression of both RTKs. In terms of prognosis, we discovered that populations with c-MET and EGFR co-expression showed a worse OS tendency compared to those with single EGFR expression or without expression, while patients with c-MET-only positivity exhibited significantly poorer overall survival, suggesting that high c-MET protein expression serves as an important adverse prognostic marker in this cohort. The co-expression subgroup showed a better OS trend relative to the isolated c-MET expression group, which seems somewhat counterintuitive given the prevailing literature, suggesting that concomitant EGFR expression could partially counteract the adverse prognostic effect conferred by c-MET. Recently emerging researches are dedicated to exploring the underlying mechanisms of this crosstalk to ensure the therapeutic effect of c-MET and EGFR combination therapy. For instance, c-MET receptor tyrosine kinase was found cross-activated by the expression of EGFR-vIII, a truncated extracellular mutant of EGFR in glioblastoma multiforme (GBM) (26). Moreover, downregulation of MET expression in CRC could prevent the HGF-dependent activation of AKT and MAPK and essentially re-sensitizes the cells to the effects of EGFR inhibitors (19). In non-small cell lung cancer (NSCLC), co-localization of c-Met and EGFR has been shown to signal through the mammalian target of rapamycin/S6 protein kinase (mTOR/S6K) pathway to have a synergistic effect on cell proliferation, downstream activation of signal transduction, and may indicate more aggressive tumor phenotype and worser prognosis (15). EGFR and MET inhibitor combination therapy have been conducted in patients with EGFR-mutant lung cancer to overcome acquired resistance of EGFR-TKIs in NSCLC (27, 28). Based on these, we hypothesize that c-MET and EGFR might engage in reciprocal cross-talk and combinatorial treatment consisting of EGFR and MET may be a breakthrough for targeting therapy in GC.

This study has several limitations that need to be acknowledged. First, this is a single-center retrospective study with inherent selection bias, and the results need to be validated in a multi-center, prospective cohort with a larger sample size to ensure a sufficiently long follow-up period, especially for the c-MET/EGFR co-expression subgroup with relatively small sample size and short follow-up time. Second, we only detected the protein expression of c-MET and EGFR by IHC, and did not detect their gene mutation, amplification or phosphorylation level, which cannot reflect the functional status of the two RTKs and the actual cross-activation of their signaling pathways. Third, the molecular mechanism of c-MET/EGFR co-expression in GC was not explored *in vitro* or *in vivo*, and the specific signaling pathway of their cross-talk needs to be further verified by cell and animal experiments. Fourth, there is no unified IHC scoring standard for c-MET and EGFR in GC, and the subjective judgment in IHC scoring may affect the results. Fifth, we did not perform stratified analysis according to Lauren classification, chemotherapy status and targeted therapy status, which may ignore the different prognostic values of c-MET/EGFR in different GC subgroups. In future studies, we will expand the sample size, combine multi-omics detection (genome, transcriptome) and *in vitro*/vivo experiments to further clarify the functional significance and molecular mechanism of c-MET/EGFR co-expression in GC.

## Conclusion

In summary, this study confirmed in a large cohort of GC patients that c-MET protein overexpression is an independent predictor of poor prognosis, which provides a potential biomarker for screening GC patients with high risk of poor prognosis. Additionally, we found a high co-expression rate of c-MET and EGFR in GC, suggesting that RTK cross-talk is a common molecular event in GC. Although the prognostic value of c-MET/EGFR co-expression needs to be further verified, the high co-expression rate provides a rational basis for exploring c-MET/EGFR combinatorial targeted therapy in GC. These findings lay a foundation for subsequent studies on the molecular mechanism of c-MET/EGFR cross-talk in GC and provide a new direction for the development of personalized targeted therapy for GC, especially for overcoming single-targeted therapy resistance.

## Data Availability

The original contributions presented in the study are included in the article/**Supplementary Material**. Further inquiries can be directed to the corresponding author.
